# Flowers of *Allium cepa* L. as Nutraceuticals: Phenolic Composition and Anti-Obesity and Antioxidant Effects in *Caenorhabditis elegans*

**DOI:** 10.3390/antiox12030720

**Published:** 2023-03-14

**Authors:** Cristina Moliner, Sonia Núñez, Guillermo Cásedas, Marta Sofía Valero, Maria Inês Dias, Lillian Barros, Víctor López, Carlota Gómez-Rincón

**Affiliations:** 1 Department of Pharmacy, Faculty of Health Sciences, Universidad San Jorge, Villanueva de Gállego, 50830 Zaragoza, Spain; 2 Instituto Agroalimentario de Aragón, IA2, Universidad de Zaragoza-CITA, 50830 Zaragoza, Spain; 3 Departamento de Farmacología, Fisiología y Medicina Legal y Forense, Universidad de Zaragoza, 50009 Zaragoza, Spain; 4 Centro de Investigação de Montanha (CIMO), Instituto Politécnico de Bragança, Campus de Santa Apolónia, 5300-253 Bragança, Portugal; 5 Laboratório Associado para a Sustentabilidade e Tecnologia em Regiões de Montanha (SusTEC), Instituto Politécnico de Bragança, Campus de Santa Apolónia, 5300-253 Bragança, Portugal

**Keywords:** edible flowers, onion, flavonoids, polyphenols, obesity, *C. elegans*

## Abstract

*Allium cepa* L., commonly known as onion, is one of the most-consumed vegetables. The benefits of the intake of its bulb are well studied and are related to its high polyphenol content. The flowers of onions are also edible; however, there are no studies about their biological properties. Our aim was to determine the polyphenolic profile and assess the antioxidant and anti-obesity capacity of an ethanolic extract from fresh flowers of *A. cepa*. The phenolic constituents were identified through LC-DAD-ESI/MSn. For the anti-obesity potential, the inhibitory activity against digestive enzymes was measured. Several in vitro assays were carried out to determine the antioxidant capacity. A *Caenorhabditis elegans* model was used to evaluate the effect of the extract on stress resistance and fat accumulation. For the first time, kaempferol and isorhamnetin glucosides were identified in the flowers. The extract reduced fat accumulation in the nematode and had a high lipase and α- glucosidase inhibitory activity. Regarding the antioxidant activity, the extract increased the survival rate of *C. elegans* exposed to lethal oxidative stress. Moreover, the activities of superoxide dismutase and catalase were enhanced by the extract. Our results demonstrate, for the first time, the antioxidant and anti-obesity activity of onion flowers and their potential use as functional foods and nutraceuticals.

## 1. Introduction

Bioactive compounds or extracts from plants have been used for health maintenance, control, and prevention of diseases since the earliest times. An example of this can be found in the management of overweight and obesity.

According to a report from the World Health Organization (WHO), overweight and obesity affect 60% of adults and are also the leading risk factors for disability [1]. They are linked to an increased risk for many non-communicable diseases, such as metabolic syndrome, osteoarthritis, or respiratory disorders, which make them a major concern for public health. Pathogenic pathways of comorbidities associated with obesity are interconnected by different factors. Especially important is oxidative stress [2]. Thus, attenuating oxidative stress is a potential therapeutic target for obesity-associated diseases.

The current approach to counteract overweight and obesity comprises lifestyle modification (nutritional and exercise interventions), and, if it is necessary, it can also include the use of drugs and bariatric surgery. Even so, in most cases, the long-term results are modest. In this context, the use of functional foods and nutraceuticals has arisen as complementary to the classic therapeutical strategy [3,4].

The patterns of food consumption are changing during the last few years. An interest in ingredients and dietary supplements that are beneficial to well-being in a manner beyond a normal healthy diet has emerged [5]. The pursuit of new functional ingredients is very challenging due to the difficulty of carrying out clinical trials to prove health benefits [6]. Therefore, several in vivo systems are used for the study of efficacy and the mechanisms involved. *Caenorhabditis elegans* has emerged as a convenient model in nutrition research. In addition to its ease of handling, there is molecular conservation in signaling pathways between invertebrates and vertebrates, making this nematode species a powerful model organism [7].

Edible flowers are promising candidates for being used as nutraceuticals or functional foods due to their rich content of bioactive compounds. Flowers are a source of polyphenols, alkaloids and carotenoids, which are non-nutritive health-promoting compounds [8]. Despite this, their use is not widespread among the general population [9], although more and more are being consumed.

*Allium cepa*, popularly called onion, is one of the most-consumed species worldwide. Several studies have been carried out to determine the composition and biological activities of the bulb and other plant parts of this species, while their flowers remain poorly studied, even though they are also edible. Different plant parts of this species have demonstrated positive results in the treatment and prevention of obesity and associated disorders, such as diabetes, hypertension and hyperlipidemia [10,11,12].

For this reason, the present study aims to determine the polyphenolic composition of *A. cepa* flowers and provide an assessment of its anti-obesity and antioxidant capacity by exploring the effect on fat accumulation and protection against oxidative stress in *C. elegans* for the first time.

## 2. Materials and Methods

### 2.1. Standards and Reagents

Acetonitrile (99.9%) was of HPLC grade and obtained from Fisher Scientific (Lisbon, Portugal). Phenolic compound standards (isorhamnetin-3-*O*-glucoside, kaempferol-3-*O*-glucoside, and quercetin-3-*O*-glucoside) were obtained from Extrasynthèse (Genay, France). Formic acid, bovine serum albumin (BSA), 2,2-diphenyl-1-picrylhydrazyl (DPPH), trolox, 2-2′-azobis(2-methyl-propionamidine)-di-hydrochloride (AAPH), 2,4,6-Tris(2-pyridyl)-1,3,5-triazine (TPTZ), xanthine, ferrous sulfate (FeSO4), CuSO_4,_ α-glucosidase from *Saccharomyces cerevisiae*, 4-nitrophenyl α-D-glucopyranoside (pNPG), lipase from porcine pancreas, 4-nitrophenyl butyrate (NPB), and bicinchoninic acid were purchased from Sigma-Aldrich (St. Louis, MO, USA). A superoxide dismutase (SOD) assay kit and catalase (CAT) assay kit were purchased from Invitrogen (Barcelona, Spain), while RIPA buffer lysis was obtained from Thermo Scientific (Madrid, Spain). 5-hydroxy-1,4-naphthoquinone (juglone) was obtained from Alfa Aesar (Ward Hill, MA, USA), and Folin–Ciocalteu reagent was purchased from Chem-Lab (Zeldelgem, Belgium). The cOmplete Protease Inhibitor Cocktail, nitroblue tetrazoluzium (NBT) and xanthine oxidase were acquired from Vidrafoc (Barcelona, Spain). All other general laboratory reagents were purchased from Panreac Química S.L.U. (Barcelona, Spain). Water was treated in a Milli-Q water purification system (TGI Pure Water Systems, Greenville, SC, USA).

### 2.2. Plant Material and Soxhlet Extraction

Fresh flowers of *A. cepa* were harvested from an organic garden in the region of Zaragoza (Spain). A total of 50 g of sample were extracted in a Soxhlet apparatus using 1 L of ethanol for 4 h. After extraction, the solvent was removed with a rotatory evaporator, and the resulting extracts were stored in the dark at −20 °C.

### 2.3. Analysis of Phenolic Compounds

The phenolic profile was determined by LC-DAD-ESI/MSn (Dionex Ultimate 3000 UPLC, Thermo Scientific, San Jose, CA, USA). These compounds were separated using a Waters Spherisorb S3 ODS-2 C18 (3 μm, 4.6 mm × 150 mm, Waters, Milford, MA, USA) column thermostatted at 35 °C and identified as previously described [13].

The obtained extracts were re-dissolved at a concentration of 10 mg/mL with the methanol:water (80:20, *v*/*v*) mixture. A double online detection was performed using a DAD (280, 330, and 370 nm as preferred wavelengths) and a mass spectrometer (MS). The solvents used were: (A) 0.1% formic acid in water and (B) acetonitrile. The elution gradient established was isocratic 15% B (5 min), 15% B to 20% B (5 min), 20–25% B (10 min), 25–35% B (10 min), 35–50% B (10 min), and re-equilibration of the column, using a flow rate of 0.5 mL/min. The MS detection was performed in negative mode, using a Linear Ion Trap LTQ XL mass spectrometer (Thermo Finnigan, San Jose, CA, USA) equipped with an ESI source.

The identification of the phenolic compounds was performed based on their chromatographic behavior and UV-vis and mass spectra by comparison with standard compounds, when available, and data reported in the literature giving a tentative identification. Data acquisition was carried out with the Xcalibur data system (Thermo Finnigan, San Jose, CA, USA). For quantitative analysis, a calibration curve for each available phenolic standard was constructed based on the UV-vis signal. For the identified phenolic compounds for which a commercial standard was not available, the quantification was performed through the calibration curve of the most similar available standard. The results were expressed as mg/g of extract.

### 2.4. In Vitro Determination of Enzyme Inhibitory Effects

#### 2.4.1. Inhibition of Pancreatic Lipase Assay

The ability of the extract to inhibit lipase was measured in 96-well plates [14]. The enzyme was diluted at a concentration of 2.5 mg/mL in 0.1 M TRIS base buffer with 5 mM CaCl_2_ (pH 7.0) and centrifugated at 2000× *g* for 7 min. A total of 40 µL of extract solution diluted in buffer, 40 µL of the enzyme, and 20 µL of buffered-substrate solution (10 mM of p-NPB) were mixed and incubated for 15 min at 37 °C. The range of extract concentrations was from 2000 to 62.5 µg/mL. Control wells were prepared by adding all reaction reagents using buffer instead of extract. Orlistat was used. Orlistat was first dissolved in ethanol at a concentration of 20 mg/mL, and afterward, serial dilutions were made in the buffer at the concentrations 1000, 100, 10, 1 and 0.1 µg/mL. Absorbance was read at 405 nm using Synergy H1 Hybrid Multi-Mode Reader (Winooski, VT, USA), and enzyme inhibition was calculated as percentage using the following Equation (1).

(1) $$Inhibition\;\left(\%\right)=\left[\;\frac{\left(Abs\;control-Abs\;sample\right)}{Abs\;control}\right]\times 100$$

#### 2.4.2. Inhibition of α-Glucosidase Assay

α-glucosidase inhibition was assessed following the procedure of Cásedas et al. [15] in a 96-well microplate reader at 405 nm. Extract and acarbose (standard) were dissolved in buffer (12.5 mM Na_2_HPO_4_ and 3.3 mM NaH_2_PO_4_; pH = 6.9) in a range of concentrations between 1000 and 31.25 µg/mL. Each well contained 50 µL of sample and 100 µL of enzyme (1 U/mL in buffer). After 10 min, 50 µL pNPG 3 mM in buffer was added and incubated at 37 °C for 20 min. Control wells contained 50 µL of buffer. Acarbose was used as positive control. Absorbance was read, and inhibition was calculated using Equation (1).

### 2.5. In Vitro Antioxidant Activity Assays

#### 2.5.1. Determination of Folin–Ciocalteu Reducing Capacity

The Folin–Ciocalteu reducing capacity was determined with the Folin–Ciocalteu method in a 96-well microplate as described by Zhang [16] with minor modifications. Briefly, Folin–Ciocalteu reagent (201 μL) was mixed with diluted extract (2.5, 5, and 10 μg/mL) in ethanol (9 μL) for 5 min in the dark at room temperature. Thereafter, 10% Na_2_CO_3_ (90 μL) was added drop by drop to the mixture. The reaction was allowed to proceed for 40 min at room temperature in darkness. The absorbance of the solution was measured at 752 nm. Pyrogallol, dissolved in ethanol, was used as the standard to prepare a calibration curve (1–0.008 mg/mL); therefore, results were expressed as mg of pyrogallol equivalents (PE)/g extract.

#### 2.5.2. DPPH Scavenging Activity

A DPPH assay was carried out following the description of Lopez [17]. The range of concentrations tested was 1000–31.25 μg/mL for the extract and 50–0.048 for ascorbic acid (standard solution). Both samples were dissolved in methanol. The reaction was initiated by adding 150 μL of DPPH dissolved in methanol (0.04 mg/mL) to 150 μL of sample dilutions. Control wells contained 150 μL of DPPH solution and 150 μL of solvent. The absorbance values were measured at 518 nm after 30 min of incubation in darkness at room temperature. The radical scavenging activity was determined as percentages according to Equation (1).

#### 2.5.3. Superoxide Radical Scavenging Activity Assay

The superoxide (O_2_^−^) radical was produced by the xanthine/xanthine oxidase system. This assay was performed according to the procedure described in the literature adapted to 96-well microplates [18]. The extract and Trolox, used as standard, were dissolved in phosphate buffer (pH = 6.9) and diluted using a twofold dilution within a final concentration range of 500–15.62 μg/mL and 100–1.56 μg/mL, respectively. A total of 30 μL of the sample were mixed with 240 μL of 22.8 µM nitroblue tetrazolium (NBT), 90 µM xanthine, and 16 mM Na_2_CO_3_ in phosphate buffer. The reaction was initiated by adding 30 μL of xanthine oxidase (168 U/L). The mixture was allowed to stand for 5 min at 37 °C, and the absorbance was measured at 560 nm. The inhibitory xanthine oxidase activity of the extract was also assayed at 295 nm. The radical scavenging activity was calculated using Equation (1).

#### 2.5.4. Ferric-Reducing Antioxidant Power (FRAP) Assay

This ferric-reducing ability of the extract was evaluated using a FRAP assay as described by Pulido [19] with minor modifications. The FRAP reagent was prepared daily and contained 10 mmol of TPTZ solution in 40 mmol/HCl, 20 mmol/L FeCl_3_·6H_2_O, and sodium acetate buffer (300 mmol/L, pH 3.6). A total of 30 μL of aqueous sample (1 mg/mL) was mixed with 90 μL of distilled water and 900 μL of FRAP reagent. The mixture was allowed to stand for 30 min at 37 °C. The absorbance was measured at 595 nm. A calibration curve was made with FeSO_4_·7H_2_O. FRAP value was expressed as μmol Fe^2+^/g extract.

#### 2.5.5. Oxygen Radical Antioxidant Capacity (ORAC) Assay

The peroxyl radical scavenging activity of the extract was estimated by ORAC assay [20]. The ORAC assay was conducted using 96 black bottom well microplates using the Synergy H1 Hybrid Multi-Mode Reader (Winooski, VT, USA). In each well, 120 μL of fluorescein (70 mM), 20 μL of a dilution of extract, methanolic Trolox (standard), or phosphate-buffered saline (PBS; blank) were placed. The reaction was started by adding 60 μL of 2,2′-Azobis(2-amidinopropane) dihydrochloride (AAPH) 12 nM. The fluorescence was measured every 70 s for 93 min at 37 °C. The area under the curve (AUC) was calculated, and the ORAC value was obtained by interpolation in a calibration curve made with Trolox (0.002–0.0016 μmol). The results were expressed as μmol Trolox equivalent (TE)/mg extract.

### 2.6. C. elegans Assays

#### 2.6.1. Strains and Maintenance Conditions

The wild-type *C. elegans* strain (N2) and *Escherichia coli* OP50 were obtained from Caenorhabditis Genetics Center (CGC, Minnesota). *C. elegans* were propagated at 20 °C on Petri dishes containing nematode growth medium (NGM) with a lawn of *E. coli* OP50 as a food source. The synchronization of worms was achieved by preparing eggs from gravid adults using an alkali-bleaching method [21].

#### 2.6.2. Assessment of Acute Toxicity

This assay was carried out following the method of Donkin and Williams with minor modifications [22]. After synchronization, wild-type worms were allowed to develop in NGM agar plates until larva stage 4 at 20 °C. At this moment, the plates were washed with K-medium (32 mM KCl, 51 mM NaCl), and the worms were re-suspended at a concentration of 80–120 worms/mL. Then, 200 μL of the worm/K-medium solution was transferred into each well of a 96-well plate. The extract was dissolved using K-medium. Fifty microliters of extract dilutions in K-medium (control) were added to the well. The survival of the worms was recorded after 24 h, and the results were expressed as a percentage of the survival rate. Approximately 40 worms per condition were tested in each assay.

#### 2.6.3. Analysis of Body Fat Accumulation in *C. elegans* Obesity Model

An obese *C. elegans* model was designed after exposing the wild-type N2 worms to an excess of 5% glucose in NGM. The conditions studied were: 5% glucose as a positive control, 5% glucose and 250 µg/mL of *A. cepa* flower extract, and plates without adding glucose as control. As a negative control substance, orlistat was used at 6 µg/mL [23]. The fat reduction obtained by this drug compared to the obese worm (positive control) was considered the maximum effect (100% reduction).

The effects of the extracts on *C. elegans* fat storages were studied by Nile Red staining and fluorimetry on the L4 stage. Synchronized L1 *C. elegans* (at least 300 individuals per condition) were grown for 48 h at 20 °C under different dietary conditions, as previously described. Total fat content was measured in nematodes by quantifying Nile Red staining images according to the previously described method [24]. This dye emits fluorescence when exposed to ultraviolet light (Nikon Intensilight C-HGFI), allowing the observation of lipids accumulated in intracellular droplets in worms. A total of 30–40 worms per condition were captured with a Nikon camera attached to an inverted Nikon Eclipse TS100 microscope after exposure to UV lightning using a GFP filter that captures 395 nm excitation and 508 nm emission wavelengths. All worms were photographed at 100× magnification and 20 s of exposure time. Images were analyzed using the image processing program ImageJ to obtain the relative fluorescence per area value of each worm.

#### 2.6.4. Evaluation of Resistance to Oxidative Stress

The oxidation stress resistance assay was based on the method described by Surco-Laos with modifications [25], using juglone to induce lethal oxidative stress. In brief, synchronized L1 worms were transferred to Petri dishes containing different concentrations of flower extract (0, 50, 100, 250 and 500 μg/mL) and were cultivated for 48 h at 20 °C. After the exposure period, the worms were washed twice with sterile water and were transferred into new wells containing 150 μM of juglone. After 24 h, the survivors were scored. At least 120 worms per condition were evaluated in each assay.

#### 2.6.5. Endogenous Antioxidant Enzymes

L1 larvae (50 nematodes/condition) were incubated in the presence of a range of concentrations of the extract (50–500 μg/mL) or in the absence of it at 20 °C. In addition, 48 h later, the nematodes were directly lysed or subjected to sublethal oxidative stress (juglone 150 μM in NGM, 1 h or 3 h) and were subsequently lysed. In order to carry out lysis, nematodes were washed twice with M9 and then mixed with RIPA buffer and cOmplete protease inhibitor cocktail. Then, the worms were disrupted by two cycles of freezing/thawing and centrifugated at 14,000× *g* 10 min at 4 °C. After centrifugation, the protein content of the supernatants was determined by the bicinchoninic acid (BCA) assays.

SOD and CAT activities were measured spectrophotometrically using commercially available kits. The activities of SOD and catalase were expressed as U/mg protein.

### 2.7. Statistical Analysis

All data come from three independent replicates. The results are reported as mean ± standard error of the means (SEM). IC_50_ values were estimated by using non-linear regression. A one-way ANOVA, Tukey’s multiple comparisons and unpaired Student’s *t*-tests were used to analyze statistical significance using GraphPad Prism version 6.0c (San Diego, CA, USA). Differences with *p* ≤ 0.05 were considered statistically significant.

## 3. Results and Discussion

### 3.1. Polyphenolic Composition of A. cepa Flowers

The extract was prepared from fresh flowers of *A. cepa* with a yield of 7.14% (mass of extract/mass of fresh flowers).

The phenolic composition of the ethanolic extract of fresh flowers of *A. cepa* was performed using an LC-DAD-ESI/MSn, and the tentative identification and quantification are presented in Table 1. Seven phenolic compounds were identified, all identified as flavonols, mainly kaempferol and isorhamnetin glycoside derivates. Peaks 5 (kaemperol-3-*O*-glucoside), 6 (isorhamnetin-3-*O*-glucoside), and 1 (kaemperol-*O*-dihexoside) were the major compounds found in the extract. The phenolic profile of this part of the sample is slightly different from those reported for the bulb and peel, in which the main compounds present were quercetin glycosides [26,27]. The quantity of kaempferol and isorhamnetin derivatives found in the extract was higher than other species of flowers, such as *Viola cornuta*, *Viola* × *wittrockiana*, or *Sambucus nigra*, but less than the value reported for *Cytisus multiflorus* [28,29].

### 3.2. In Vitro Inhibition of α-Glucosidase and Pancreatic Lipase

The antiobesogenic potential was first evaluated through the in vitro inhibition of the lipase and α-glucosidase enzymes. IC_50_ values of samples and control substances (acarbose and orlistat) are presented in Table 2.

*A. cepa* flower extract showed α-glucosidase inhibition and pancreatic lipase inhibition; the percentages of inhibition were not as high as the control substances, but this activity should be taken in consideration.

Through the targeting of these digestive enzymes, the absorption of sugars and lipids can be reduced or controlled and therefore be useful in the management of parameters related to obesity, cardiovascular diseases, and diabetes. As stated by the European Medicines Agency [30], *A. cepa* has been seldom traditionally used to treat diabetes, but there are reports of its antihyperglycemic activity, cardiovascular benefits, and lipid-lowering effects.

Various studies have shown that onion extracts can exhibit inhibitory activity against the enzymes connected to metabolic syndrome and oxidative stress [31,32,33,34]. There are precedents for the inhibition of lipase and α-glucosidase by diverse parts of *A. cepa,* such as leaves [35], skin [34], and pulp [36], but to the best of our knowledge, this is the first time that these bioactivities have been proven in the flower extract. For lipase inhibition, the obtained IC_50_ value was lower than those described for the skin extract (53.70 mg/mL) or bulb juice (9.5 mg/mL) [31,35]. The IC_50_ value for α-glucosidase inhibition was higher; Nile et al. found an IC_50_ of 55.2 μg/mL for an ethanolic extract of solid onion waste (basal and apical trimming and outer skin parts) [33]. Our results suggest that *A. cepa* flower extract may serve as a potential source of natural lipase and α-glucosidase inhibitors.

### 3.3. In Vitro Antioxidant Activity

*A. cepa* flower extract was first tested in vitro through several antioxidant assays. The results are summarized in Table 3.

DPPH and O_2_^−^ assays were used to assess the radical scavenging activity of the extract. *A. cepa* extract exhibited moderate activity in the DPPH assay. The maximum percentage of inhibition of DPPH was 94% ± 1 at 1000 μg/mL. A higher power was found to neutralize the superoxide radical. This is an important fact because this radical is one of the most prevalent ROS in biological systems [37].

Beyond scavenging oxidants, the ability to perform reduction, especially of Fe^2+^, is also considered an important antioxidant mechanism [38]. To evaluate this, FRAP and Folin–Ciocalteau assays were carried out. The FRAP value was 6 ± 2 mmol Fe^2+^/g extract, while the reducing capacity quantified by the Folin–Ciocalteau method was 17 ± 2 mg PE/g extract. This method is known as a measure of the total phenolic content; however, due to the non-specificity of the reaction, it is more valuable to assess the total antioxidant-reducing activity. The Folin–Ciocalteau reagent is not only reduced by phenolic compounds, such as flavonoids; other compounds can also react against it, such as vitamins or proteins [39]. As a consequence, there is an overestimation of the total phenolic content compared with the value obtained with LC-DAD-ESI/MSn.

ORAC is one of the main assays to assess the hydrogen atom transfer of extracts. The ORAC value of the flower extract was 1 ± 0.1 μmol TE/mg extract. Xiong et al. determined the ORAC value of acetone extracts from 10 common edible flowers, and most of them showed a lower value than our data [40].

The comparison of our results with others reported in the literature about other parts of *A. cepa* is difficult due to the use of different units and methods for the antioxidant activity evaluation. These studies have shown a strong antioxidant activity of the skin, pulp and essential oil [41,42,43,44]. Our findings show promising activity also for the flowers of this species.

### 3.4. C. elegans Assays

In order to better understand the biological effects of this extract, the antioxidant and anti-obesity activity was assessed in *C. elegans,* as this nematode offers the possibility of detecting phenotypic changes.

#### 3.4.1. Assessment of Acute Toxicity of Fresh Flowers

**Figure 1:**
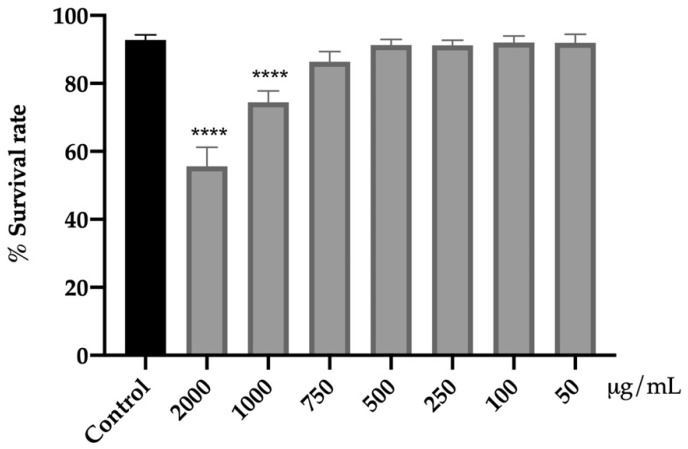
Effect of the extract from fresh flowers of *A. cepa* on *C. elegans* N2 viability. Results are represented as mean ± SEM. Differences compared to the control group were considered significant at *p* < 0.0001 (****).

Initially, the effect of the extract on the viability of *C. elegans* N2 was carried out to evaluate its acute toxicity and to establish the range of non-toxic concentrations (Figure 1).

The range of concentrations of 50–750 μg/mL for 24 h did not affect viability as compared to the control group. However, the higher concentrations tested (1000 and 2000 μg/mL) had a negative impact on the viability of the nematodes (*p* < 0.0001). The mortality rate was increased by 19% (1000 μg/mL) and 37% (2000 μg/mL) with respect to the control without reaching the lethal dose 50 (LD_50_).

The nematicide power of the flower extract is scarce. This is the first time that the impact on *C. elegans* viability of *A. cepa* flowers has been described. No other studies have been found that evaluate this activity in other nematode species; however, there are two studies about the nematicidal action. One of them was carried out using onion oil, while the other one tested two purified oligosaccharides from onion bulb extract [45,46]. As commented previously, there is a difference in the phytochemicals present in different parts of the plant, and these differences also depend on the extraction technique.

#### 3.4.2. *A. cepa* Flower Extract Decreased Fat Accumulation

*C. elegans* is a great model for exploring lipid metabolism because the regulatory pathways of energy homeostasis are highly conserved between mammals and this nematode. Numerous studies have shown that *C. elegans* is an excellent tool in the search for bioactive compounds that allow modulating lipid metabolism, contributing to the control of obesity [47,48,49].

**Figure 2:**
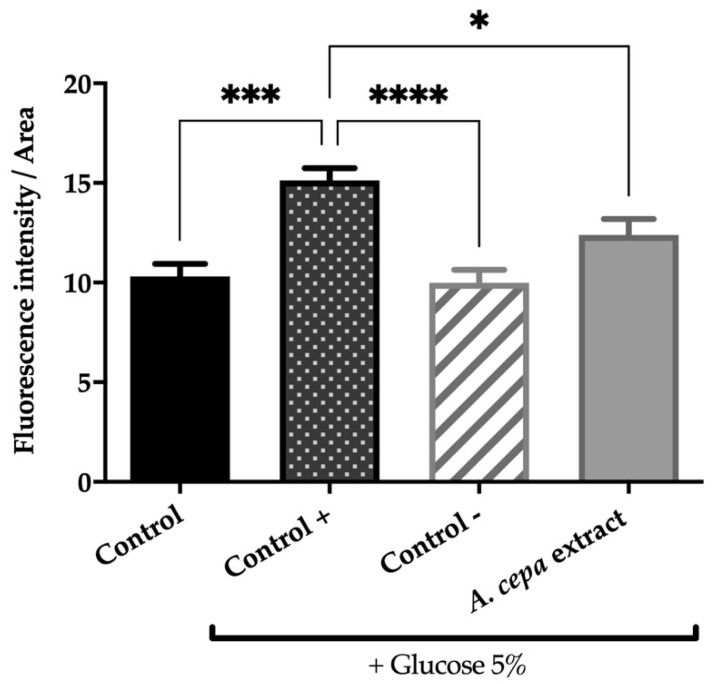
Effect of the extract from fresh flowers of *A. cepa* on *C. elegans* N2 total fat. Control group was nematodes without supplementation of glucose, while *A. cepa* extract (250 µg/mL), control + (orlistat, 6 µg/mL) and control groups were cultivated in the presence of 5% glucose. Results are represented as mean ± SEM (n = 50–60 worms). Differences were considered significant at *p* ≤ 0.05 (*), *p* < 0.001 (***) and *p* < 0.0001 (****).

As can be seen in Figure 2, glucose supplementation increases fat deposits in obese control worms by 31.8% compared to control animals (*p* < 0.001), validating the designed in vivo model. On the other hand, our results show that treatment with orlistat, the reference drug, or the extract of *A. cepa* flowers effectively reverses this effect. The dietary addition of 250 μg/mL of *A. cepa* flower extract reduced the fat deposits in the treated worms by 18.2% compared to the obese control without treatment (*p* < 0.05). A similar effect was observed in the group treated with orlistat, the reference drug, which produced a reduction in lipid content of 34.6% (*p* < 0.0001). For both treatments, no statistical differences were achieved in the non-obese worms (control group), which suggests that onion flower extract, as orlistat, allows the reduction of fatty deposits to physiological levels in the *C. elegans* N2 strain.

Energy homeostasis is a highly complex phenomenon whose regulation is influenced by numerous factors. In *C. elegans*, more than 400 genes involved in the maintenance of body reserves have been described. Many of these pathways are highly conserved in humans, so in recent years, a considerable number of authors have used this model in the study of fat metabolism and obesity [48]. Recent research has shown that phenolic compounds can reduce fatty deposits in *C. elegans* by increasing lipolysis or reducing lipogenesis through different mechanisms controlled by several genetic pathways. The in vivo obesity model used in this study does not allow the identification of a specific mechanism of action for the decrease in total fat observed in worms treated with *A. cepa*. However, the identification in the extract of kaempferol and isorhamnetin derivates as major components suggests that these compounds, whose anti-obesity effect has been previously described, could be responsible for the observed activity [47,48]. Thereby, Farrias-Pereira et al. showed that isorhamnetin reduces fat accumulation in *C. elegans* by increasing fat oxidation [48]. This effect was dependent on the nhr-49 pathway, which is involved in fatty acid β-oxidation and lipolysis. As is well known, fat accumulation increases oxidative stress damage, and on the contrary, an increase in ROS production leads to an excessive accumulation of fat [50]. The clear antioxidant effect shown by the extract could contribute to the observed anti-obesity effect.

#### 3.4.3. Onion Flower Extract Attenuates the Oxidative Stress Toxicity Induced by Juglone

**Figure 3:**
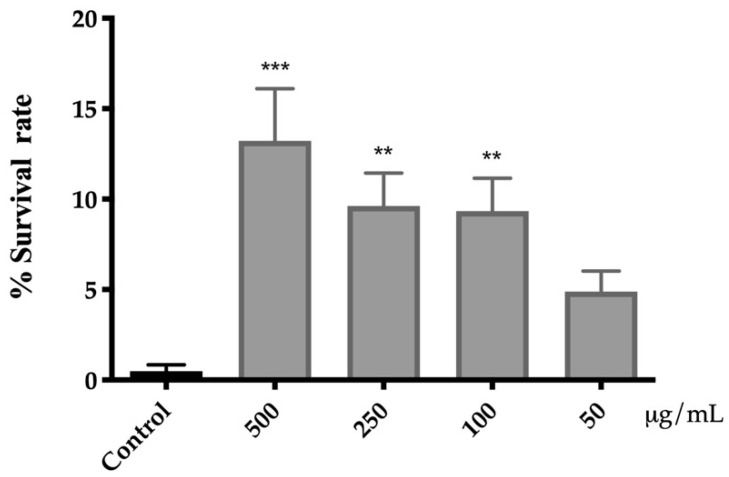
Effect of *A. cepa* flower extract on the response to lethal oxidative stress induced by juglone on *C. elegans*. Results are represented as mean ± SEM. Differences compared to control group were considered significant at *p* < 0.01 (**) and *p* < 0.001 (***).

The protective activity of the extract of *A. cepa* flowers against oxidative stress was evaluated by exposing *C. elegans* to a lethal dose of juglone. Juglone is a powerful pro-oxidant, which increases the generation of intracellular superoxide radicals that can damage cellular components [51]. As shown in Figure 3, the pre-treatment with *A. cepa* significantly increased the survival rate of nematodes, protecting them from oxidative stress. The best response was found in the group treated with 500 µg/mL of flower extract, for which the survival rate was increased from 0.4% ± 0.3 (control group) to 13% ± 3.

Our results reveal a protective potential for *A. cepa* flowers against oxidative stress, which is in concordance with the in vitro assays described above and the phenolic composition. To the best of our knowledge, this is the first study of the antioxidant potential of *A. cepa* using *C. elegans*. The polyphenols present in the extract have shown a protective effect against oxidative stress on this model organism. Kampkötter et al. showed that kaempferol decreases the accumulation of ROS and oxidative stress [52]. Similar findings were also made in assays performed with isorhamnetin, which increased the survival rate of nematodes after juglone exposure by around 15% [53].

#### 3.4.4. Impact of the Extract on Endogenous Antioxidant Enzyme Activities

The antioxidant enzymes catalase (CAT) and superoxide dismutase (SOD) are the main defense system against oxidative injury. The inductions of these antioxidant enzymes are potential pharmacology targets to attenuate ROS-induced damage related to cardiovascular or neurodegenerative disorders [54]. Both enzymes are highly conserved in the nematode *C. elegans* [55].

**Figure 4:**
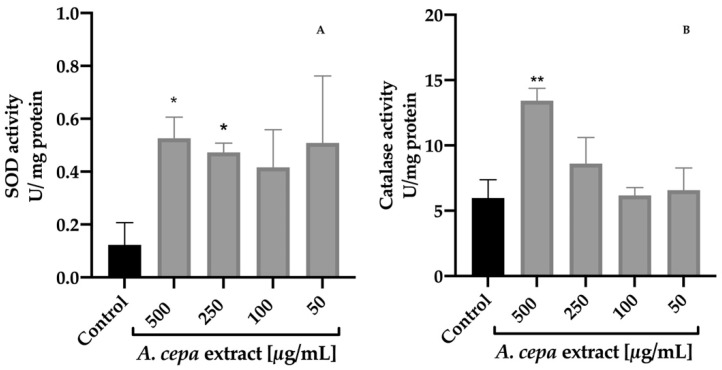
Effect of *A. cepa* flower extract on SOD (**A**) and CAT (**B**) activity on *C. elegans*. Results are represented as mean ± SEM. Differences compared to control group were considered significant at *p* ≤ 0.05 (*) and *p* < 0.01 (**).

The effect *of A. cepa* extract on SOD and CAT activities was determined on *C. elegans* (Figure 4). After 48 h of treatment with the extract, the results revealed a significant increase in the activity of both enzymes with respect to the control group. The best response was found in the group treated with 500 µg/mL leading to a 4.3 and 2.3-fold increment for SOD and CAT activity compared to the control worms, respectively. The 250 µg/mL group also increased SOD activity (3.8-fold) without having a significant effect on the CAT activity against the control group.

**Figure 5:**
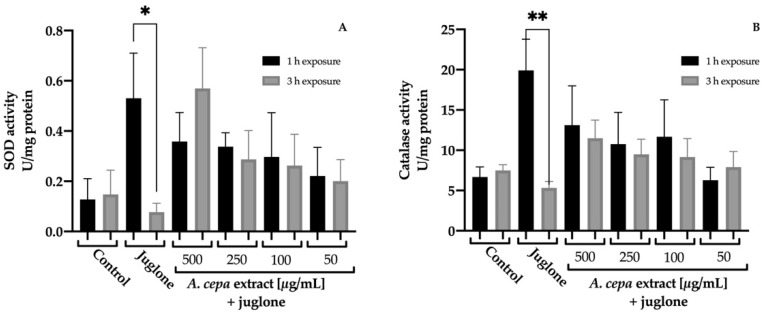
Effect of *A. cepa* flower extract on SOD (**A**) and CAT (**B**) activity after sublethal oxidative stress induced by exposure *C. elegans* to juglone for 1 o 3 h. Results are represented as mean ± SEM. Differences were considered significant at *p* ≤ 0.05 (*) and *p* < 0.01 (**).

To deepen the protective effect of the extract against juglone damage, the activity of these endogenous antioxidant enzymes was determined after inducing a sublethal oxidative stress. In order to observe the differences over time, worms were exposed to juglone 150 μM for 1 h or 3 h, and the results are shown in Figure 5. In both cases, the exposition to juglone for 1 h causes a significant increase in their activity to counteract the free radicals generated by juglone. However, after 3 h of exposure, the assessment showed a significant reduction in SOD and CAT. This fact was not observed in the nematodes treated with the extracts, in which no statistical differences in enzymatic activity between the different times were found. CAT and SOD levels remained constant. This could be related to the positive effect on the survival rate of nematodes exposed to a lethal oxidative stress. However, no statistical differences were found between the different groups in the same exposure time.

Previous studies found similar effects in SOD and CAT activities of other parts of the plant using cell cultures, rats, and rabbits [56,57,58]. These enzymes are biomarkers of antioxidant defenses of the organisms.

*A. cepa* flowers exhibit a protective effect against juglone toxicity supported by in vitro assays. However, the results obtained from the endogenous enzymes are insufficient to explain the implied antioxidant mechanism, which should be further clarified in the future.

The use of functional foods and nutraceuticals as non-pharmacological interventions has a great potential for reducing obesity, oxidative stress, and associated comorbidities. Certain clinical trials are using *A. cepa* as a dietary intervention, such as the study performed by Jeon et al.; in this study, overweight South Korean patients received supplementation with steamed onion, which caused a decrease in total body fat and an improvement in metabolic parameters [12]. These encouraging results support the need to continue the research on the different edible parts of onions and related vegetables.

## 4. Conclusions

We demonstrated, for the first time, the potential use of onion flowers as a functional food due to the presence of polyphenols and their antioxidant and anti-obesity activities exhibited in vitro and in vivo. *A. cepa* flowers are a rich source of kaempferol and isorhamnetin glycoside derivatives. The extract has demonstrated lipase and α-glucosidase inhibiting properties, as well as the ability to mitigate the fat accumulation and toxicity induced by oxidative stress in *C. elegans*. This work put forward the idea of using certain flowers of plant foods as edible, healthy ingredients with health-promoting properties.

## Figures and Tables

**Table 1:** Retention time (Rt), wavelengths of maximum absorption in the visible region (λ_max_), mass spectral data, tentative identification, and quantification (mg/g of extract) of the phenolic compounds present in *A. cepa* flowers.

Peak	Rt (min)	λ_max (nm)_	Molecular Ion [M-H]^−^ (*m/z*)	MS^2^ (*m/z*)	Tentative Identification	Qauntification (mg/g of Extract)
**1**	14.46	350	609	447 (72), 285 (100)	Kaempferol-*O*-dihexoside	0.887 ± 0.001
**2**	16.44	346	609	429 (100), 285 (73)	Kaempferol-*O*-dihexoside	0.52 ± 0.01
**3**	18.38	341	593	285 (100)	Kaempferol-3-*O*-rutinoside	0.276 ± 0.001
**4**	21.27	343	609	285 (100)	Kaempferol-*O*-dihexoside	0.447 ± 0.001
**5**	22.39	347	447	285 (100)	Kaempferol-3-*O*-glucoside	1.12 ± 0.01
**6**	23.34	353	477	315 (100)	Isorhamnetin-3-*O*-glucoside	0.93 ± 0.01
**7**	24.23	317	623	477 (10), 315 (100)	Isorhamnetin-*O*-coumaroylhexoside	0.333 ± 0.004
					Total phenolic compounds	4.50 ± 0.01

**Table 2:** Anti-obesity activity of flower extract. The results are presented as mean ± SEM.

Sample	α-Glucosidase IC_50_ (μg/mL)	Lipase IC_50_ (μg/mL)
*A. cepa* flower extract	412.1 ± 0.4	677.1 ± 68.4
Acarbose	297.2 ± 15.8	-
Orlistat	-	27.7 ± 13.3

-, non-assessed.

**Table 3:** Antioxidant and reducing activity of flower extract. Results are presented as means ± SEM.

Assay	DPPH IC_50_ (μg/mL)	O_2_^−^ IC_50_ (μg/mL)	Folin-Ciocalteau mg PE/g Extract	FRAP mmol Fe^2+^/g Extract	ORAC μmol TE/mg Extract
*A. cepa* flower extract	471 ± 46	229 ± 39	17 ± 2	6 ± 2	1 ± 0.1
Ascorbic acid	1.5 ± 0.1	-	-	-	-
Trolox	-	28 ± 1	-	-	-

mg of pyrogallol equivalents/g extract; mg of PE/g extract; μmol trolox-equivalent/mg extract; μmol TE/mg extract; -, non-assessed.

## Data Availability

Data are contained within the article.
